# Hereditary angioedema (HAE) in children and adolescents: New treatment options 

**DOI:** 10.5414/ALX02532E

**Published:** 2024-10-30

**Authors:** Maria Fasshauer, Bettina Wedi

**Affiliations:** 1 Klinikum St. Georg Leipzig, Clinic for Pediatrics and Adolescent Medicine, ImmunDefektCentrum Leipzig (IDCL), Leipzig, and; 2 Hannover Medical School (MHH), Department of Dermatology, Allergology and Venereology, Interdisciplinary Allergy Center of the MHH, Treatment Center for Hereditary Angioedema of the MHH, Hannover, Germany

**Keywords:** hereditary angioedema (HAE), children and adolescents, on-demand treatment, long-term prophylaxis, plasma kallikrein and prekallikrein inhibition, factor XIIa inhibition, bradykinin B2 receptor (BK2R) inhibition, gene editing, quality of life

## Abstract

Modern management of hereditary angioedema (HAE) due to reduced C1 inhibitor (C1-INH) function or concentration (HAE-C1-INH) focuses on individualized therapeutic strategies to address the specific needs of children and adolescents as well as the severity of the disease. Psychosocial factors such as the burden of disease and therapy on quality of life and participation play an important role. New medications have already significantly improved the prognosis and health related quality of life in HAE patients, but not all of these therapies have yet been approved for children. Further treatment options that inhibit bradykinin effects are currently being investigated. They target factor XIIa, prekallikrein, plasma kallikrein, or the bradykinin B2 receptor. Modern research focuses on oral options or long-acting parenteral therapy approaches to further optimize care and, in particular, the needs of children. There are also initial developments in the field of gene therapy, which could represent a causal treatment option for HAE in the future. This article focuses on the presentation and treatment of HAE type I (reduced C1-INH concentration) and HAE type II (impaired C1-INH function) in children and adolescents. Acquired AE and HAE with normal C1-INH are rare in the pediatric age group and are not discussed in detail here.

## Introduction 

Hereditary angioedema (HAE) is a rare autosomal-dominant inherited disease caused by a deficiency or reduced function of the C1 esterase inhibitor (C1-INH). This leads to uncontrolled swelling (angioedema) of the skin and mucous membranes, which can be disfiguring, painful, and life-threatening (laryngeal swellings). In the last 10 years, therapeutic options for HAE-C1-INH have significantly improved due to a better understanding of the pathomechanisms. Treatment goal is to control swelling attacks, minimize disease burden, and improve quality of life. This article is based on the international WAO/EAACI guideline from 2021 [1] as well as the revised S1 guideline from 2019 [2], and especially on the 2020 update of recommendations for the treatment of HAE in children and adolescents in the German-speaking region [3]. Modern, individualized therapies have already been established for adults and adolescents. The specific needs of children are increasingly being addressed, with current research focusing on orally available substances or parenteral preparations with long-lasting effects. This article highlights treatment options approved in Germany, considering the privileged healthcare situation in industrialized countries, and provides insights into current therapeutic approaches based on the latest scientific findings. 

## Pathogenesis 

**Figure 1.**
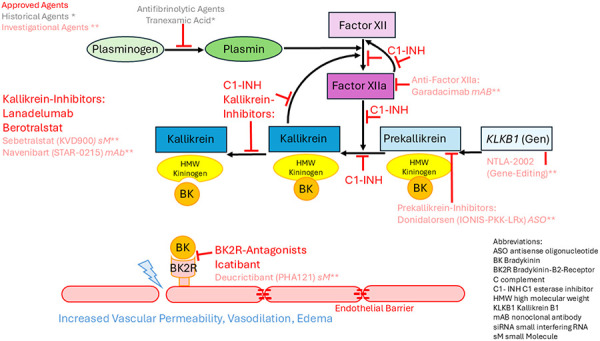
Pathogenesis of hereditary angioedema and therapeutic approaches, considering historical* and investigational** agents. Adapted with permission from Volker Wahn from [3] based on [5] and [6].

HAE is one of the rare bradykinin-mediated angioedemas. About 85% of patients have reduced C1-INH concentration (HAE-C1-INH type I), and about 15% have normal (or elevated) levels with reduced C1-INH activity (type II). Both types exhibit similar symptoms. The disease is inherited in an autosomal-dominant manner in 75 – 80% of affected individuals, while the remaining cases result from de novo variants. These mutations are located in the SERPING1 gene on chromosome 11 (11q12-q13.1), which encodes C1-INH. C1-INH is primarily produced in the liver and is the main inhibitor of active proteases in the contact activation system, plasma kallikrein, and the coagulation pathway, including activated factor XII. A deficiency of C1-INH leads to dysregulation of the contact and kinin systems, resulting in an overproduction of bradykinin, which primarily increases vascular permeability by binding to the bradykinin B2 receptor and promotes the development of angioedema (Figure 1) [4]. 

Men and women are equally affected by HAE-C1-INH and there are no known differences between various ethnic groups. The majority of patients develop first HAE symptoms during childhood or early adolescence with a median age of approximately 11 years [2]. Recently collected retrospective prescription data of HAE medications in children and adolescents in Germany between 2016 and 2021 suggest an HAE prevalence of 2.51 : 100,000 in pediatric patients aged < 12 years in Germany. The overall cohort’s prevalence was 3.35 per 100,000 which is higher than the previously published average prevalence of 1 – 2 : 100,000 but within the range of reported values from 0.67 – 10 : 100,000 [7]. 

## Symptoms 

**Figure 2.**
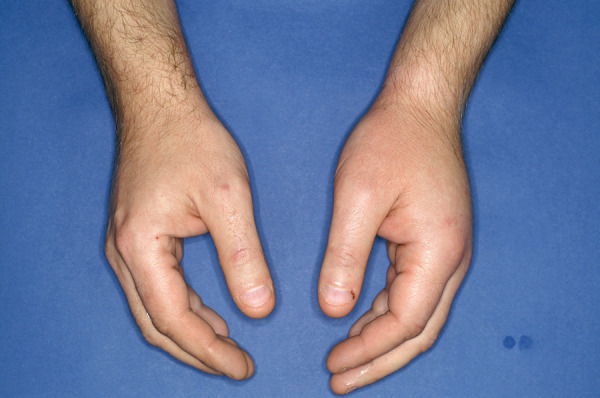
HAE swelling of the left hand.

**Figure 3.**
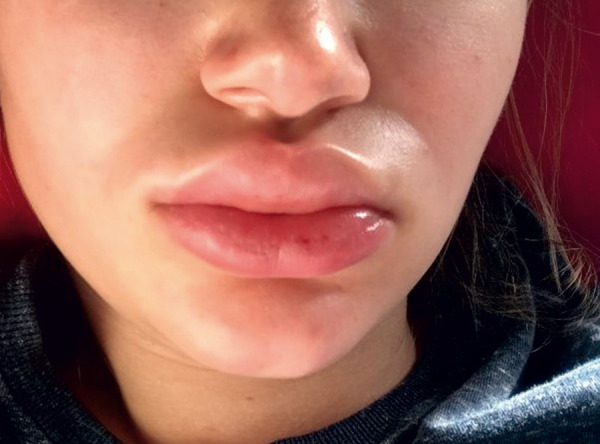
HAE swelling of the lip.

**Figure 4.**
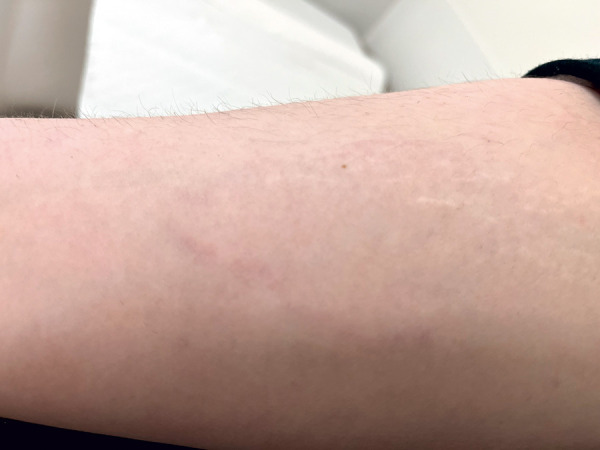
Erythema marginatum on the forearm preceeding an HAE swelling.

HAE symptoms typically manifest in childhood or adolescence but are extremely rare in newborns and infants. However, early onset often indicates a more severe course. During puberty and adolescence, the frequency and severity of HAE attacks may increase [1]. First symptoms often include recurrent abdominal pain accompanied by nausea, vomiting, and/or diarrhea, caused by angioedema of the intestinal mucosa. Other common symptoms include peripheral swelling of the skin, i.e., of the extremities (Figure 2), face (Figure 3), thoracic wall, or genital area, which are localized, doughy-firm, and skin-colored. These swellings can also be painful and differ from histamine-mediated angioedema by the absence of urticaria and itching. In individual cases, angioedema may be preceded by erythema marginatum, a non-itching, sharply defined (not raised!) reddening of the skin (Figure 4). This symptom is more common in children and occurs in about 50% of cases. Misinterpreting it as urticaria may lead to misdiagnosis and ineffective treatment. Other prodromal symptoms of an HAE attack may include fatigue, exhaustion, thirst, aggression, or depressive mood [1, 2, 3]. 

HAE swellings develop slowly and are usually self-limiting within 2 – 5 days. Triggers can be trauma, infections, hormonal changes (such as menstruation), medications like ACE inhibitors or estrogen preparations as well as emotional stress. Medical procedures in the mouth or throat can trigger life-threatening swelling of the upper airways, although this usually does not occur during the procedure itself but with a delay of a few hours [1, 2, 3]. HAE attacks manifesting with severe abdominal pain only and without skin symptoms can lead to unnecessary surgeries because of suspected acute abdomen [2]. The symptoms of HAE-C1-INH are not specific and may be confused with those of more common conditions. However, early diagnosis is crucial to initiate appropriate therapeutic measures and prevent life-threatening complications [3]. 

## Diagnostics 

Patients suspected of having HAE-C1-INH based on clinical symptoms or a positive family history should undergo testing. Early differentiation between HAE and other types of angioedema, such as histamine- or drug-induced angioedema, is crucial to initiate appropriate treatment [1, 3]. Laboratory diagnostics involve evaluating C1-INH concentration and activity, as well as C4 concentration in plasma. A “screening test” that examines only one of these parameters is insufficient to rule out HAE-C1-INH [1, 2, 3]. In HAE type I, C1-INH activity and concentration as well as C4 are reduced, while in HAE type II only C1-INH activity and C4 are reduced, while C1-INH concentration is normal or may even be elevated (Table 1) [1, 2, 3]. 

Abnormal test results should ideally be confirmed in certified laboratories. Genetic testing is not recommended as a routine examination but can support the diagnosis in doubtful cases, atypical presentations, or in very young infants or when distinguishing types of HAE with normal C1-INH levels [1, 2, 3]. 

Due to the autosomal-dominant inheritance of HAE-C1-INH, there is a 50% chance that offspring of affected individuals will inherit the condition. These children are considered potentially affected until HAE-C1-INH is ruled out and must be monitored closely and tested as early as possible, ideally before clinical symptoms appear. Testing for C1-INH in very young infants should be repeated at the age of 1 year [1]. 

Diagnosing HAE for the first time in children is challenging, as symptoms such as abdominal pain often resemble those of other, more common pediatric conditions. An abdominal ultrasound during the acute phase of an HAE attack may show intestinal wall thickening, but a normal abdominal ultrasound does not necessarily exclude HAE [3]. Recurrent abdominal pain and peripheral swelling, particularly with a positive family history, may indicate HAE [1, 3]. 

## Therapeutic approaches 

Children and adolescents with HAE-C1-INH should be treated by medical personnel experienced in HAE management or in specialized centers. Like adults, they require an emergency document and a regularly updated treatment plan tailored to the child’s life circumstances and medical needs. Medical decisions are made together with the families and consider the child’s needs. Treatment includes both on-demand therapy for acute attacks as well as prophylactic strategies, with various medications available for different indications and ways of application, although not all are approved for every age group (Tables 2, 3, 4) [3]. 

### On-demand therapy 

This most frequently used form of treatment is usually initially carried out by a physician. Ideally, patients or caregivers (after appropriate training) can also administer medication at home. In general, on-demand therapy should be considered for every attack. Treatment should begin at the first signs of an attack to positively influence severity and course. This is especially important for swellings of the face, mouth, or tongue, which can lead to a laryngeal attack, a medical emergency that must be treated immediately (Table 2) [3]. 

**C1 inhibitor concentrates**

This substitution therapy replaces the missing or inadequately functioning C1-INH (Figure 1) and over the years extensive experience with its efficacy and safety has been accumulated. C1-INH concentrates are administered intravenously during an acute HAE attack, and studies have shown that they are effective and safe also for children. Both, human plasma-based and recombinant C1 inhibitors are available [3]. 

*Human plasma-derived C1 inhibitor concentrates (pdC1-INH)*

The indication-specific use of Product A (500 or 1,500 IU) and Product B (500 IU) is shown in Table 2 [3, 8]. 

*Recombinant C1 inhibitor (rhC1-INH) – Conestat α*

Conestat α is a recombinant analogue of the human C1 esterase inhibitor (rhC1-INH), produced via DNA technology in the milk of transgenic rabbits, and has an amino acid sequence identical to endogenous C1-INH. It is administered intravenously in weight-adapted doses to children aged 2 years and older. Efficacy and safety results in children and adolescents are comparable to those in adults [9]. 

**Bradykinin B2 receptor antagonist**

Icatibant is a bradykinin B2 receptor antagonist that reduces the effect of bradykinin on blood vessels, preventing further fluid leakage and promoting edema resolution (Figure 1). Since 2017, Icatibant has been approved for children and adolescents aged 2 – 17 years for on-demand treatment of HAE attacks. The dosage is weight-adapted and can easily be administered subcutaneously with a dosing aid – a graduated syringe (in 0.5 mL increments) – that helps to measure the exact dose needed for the child in amounts from 1 mL up to 3 mL. In case of insufficient response, intravenous administration of a C1-INH concentrate may be necessary [10]. 

### Short-term prophylaxis 

Short-term prophylaxis refers to C1-INH replacement prior to surgical or invasive medical procedures, especially in the head-neck area, to prevent an HAE attack due to mechanical irritation. Intravenously administered plasma-derived C1 inhibitor concentrates are approved for this purpose (Table 3) [8]. Patients receive an individualized treatment plan in written form prior to medical interventions [3]. 

### Long-term prophylaxis 

Long-term prophylaxis is a maintenance therapy aimed at preventing attacks or significantly reducing the frequency of attacks, particularly in severe cases of HAE (Table 4). Long-term prophylaxis is gaining therapeutic importance to reduce the psychological effects of HAE, such as anxiety and depression, improve patients’ quality of life, and enable normal participation in daily activities [1, 3]. 

**Human plasma-derived C1 inhibitor concentrates (pdC1-INH)**

C1-INH concentrates can be used not only for on-demand treatment or short-term prophylaxis but also as maintenance prophylactic therapy to reduce the frequency and severity of HAE attacks. Regular intravenous administration of C1-INH (Product B) [11] or subcutaneous administration of C1-INH (Product A) [12] has been shown to be effective, safe, and beneficial in minimizing HAE attacks and improving the quality of life also in children (Table 4). 

**Plasma kallikrein inhibition**

Modern therapeutic approaches in long-term prophylaxis target the pathogenesis of the disease by reducing the effects of kallikrein in the plasma, thereby limiting the bradykinin effects on blood vessels. 

Lanadelumab is a monoclonal antibody that inhibits the activity of plasma kallikrein and reduces it to the level of healthy individuals when administered subcutaneously every 2 weeks. Lanadelumab is now approved for long-term prophylaxis of HAE also in children aged 2 years and older, in weight-adapted doses. Clinical studies have demonstrated that it significantly reduces the frequency of HAE attacks in children and adolescents and is well tolerated (Table 4) [13]. 

Berotralstat became the first oral kallikrein inhibitor and was introduced in 2021. This drug binds to plasma kallikrein as an enzyme inhibitor, reducing the cleavage of high-molecular-weight kininogen and consequently, the release of bradykinin. It is approved for adolescents aged 12 years and older as well as for adults (Table 4) [14]. Currently, the APeX-P phase 3 study is investigating the safety and pharmacokinetics of berotralstat prophylaxis in children with HAE aged 2 to < 12 years. 

## General treatment concepts and specific considerations for children 

Patients and their caregivers should be well informed about HAE, possible triggers (such as certain medications), and should be equipped with emergency documents. It is recommended that a swelling calendar (or app) be kept to document HAE attacks, prodromal symptoms, triggers, and the administered medication. The therapeutic options described in this article provide modern and effective ways for children and adolescents with HAE for both on-demand treatment and prophylaxis. In cases of bradykinin-mediated HAE attacks, ineffective medications such as corticosteroids, antihistamines, and epinephrine, which are effective in histamine-mediated angioedema only, should not be used [3]. Regular medical check-ups are necessary to monitor the frequency and severity of attacks, assess treatment efficacy, and detect potential side effects early. The treatment plan should be continuously reviewed and updated. The possibility of home therapy should also be pointed out and appropriate training provided. Medication dosages in children are regularly adjusted based on weight and/or age. Precise written advice concerning short-term prophylaxis should also be provided [1, 3, 15]. 

Therapeutic success and quality of life should regularly be recorded and evaluated. Validated questionnaires such as the Angioedema Control Test (AECT) [16] and the Angioedema Quality of Life Instrument (AE-QoL) [17] are available for adults, but there are no validated instruments for children or adolescents yet. HAE can have significant emotional and social impact on affected children and adolescents. Fear of attacks or pain, as well as daily limitations or embarrassment due to disfiguring angioedema, can lead to psychological stress [18]. A strong patient organization, such as Hereditary Angioedema e.V. (HAE e.V.), supports individuals with HAE and their families in Germany and can be found at https://hae-online.de/. A holistic, multidisciplinary approach is recommended for treating HAE patients, which should include psychological support and counseling for the entire family by medical professionals experienced in HAE diagnosis and treatment [3]. 

## Current developments and future outlook 

New drugs have also recently been approved for pediatric HAE patients. However, there is still a need for oral therapies or parenteral options with longer dosing intervals in HAE treatment for children. Several novel agents for on-demand treatment, but also for long-term prophylaxis, are currently being investigated regarding these questions. The improved understanding of HAE pathogenesis is currently enabling the rapid development of new therapeutic strategies aimed at inhibiting bradykinin effects. These therapies target factor XIIa, prekallikrein, plasma kallikrein, or the bradykinin B2 receptor (Figure 1, marked with **) [6]. 

### Factor XIIa inhibition 

Garadacimab (CSL312): Studies are underway to investigate the use of a factor XIIa inhibitor (garadacimab) in long-term prophylaxis. Initial positive data have been reported on its subcutaneous administration every 4 weeks in long-term prophylaxis for HAE in adults and adolescents [19]. At the EAACI Congress 2024, the start of a phase 3 study investigating garadacimab for the prophylactic treatment of pediatric patients (aged 2 – 11 years) with HAE was announced. 

### Prekallikrein inhibition 

Donidalorsen (IONIS-PKKRx) is an antisense oligonucleotide that binds to the mRNA of plasma prekallikrein, inhibiting kallikrein formation. Treatment with donidalorsen reduced the frequency of HAE attacks, supporting its potential prophylactic use in hereditary angioedema [20]. 

### Plasma kallikrein inhibition 

Sebetralstat (KVD900): For on-demand treatment, there are current developments for orally available medications, which are particularly interesting for children. Sebetralstat (KVD900) is an oral small molecule that selectively inhibits plasma kallikrein as well as the formation of factor XIIa and the activation of plasma prekallikrein. As a result, the activation of the contact system is blocked for up to 6 hours, making it suitable for on-demand treatment of acute HAE attacks. First positive study results in adolescent and adult patients with HAE type I or II in the KONFIDENT study have already been reported [21, 22]. Recently, the start of the KONFIDENT-KID study was announced, investigating sebetralstat in children aged 2 – 11 years (https://clinicaltrials.gov/study/NCT06467084). 

Navenibart (STAR-0215) is a long-acting monoclonal antibody targeting activated kallikrein. In a phase 1a study with increasing single doses, the safety, pharmacokinetics, and pharmacodynamics of this substance were investigated. The drug is administered subcutaneously and is a potential agent for long-term prophylaxis that can be administered every 3 months or less frequently [23]. 

### Bradykinin B2 receptor (BK2R) inhibition 

Deucrictibant (PHA121) is an investigational oral drug developed for on-demand treatment of HAE attacks as well as for the prophylactic use in HAE-C1-INH. It is a small molecule that blocks the bradykinin B2 receptor and is being tested in the form of an oral suspension, an immediate-release capsule, or an extended-release tablet. A global phase 3 study is planned https://angioedemanews.com/pha121/.

### Gene editing 

NTLA-2002 (CRISPR-Cas9 Gene Editing) is an investigational drug based on “in vivo gene editing”. The approach of permanently correcting the genetic defect in HAE could potentially provide a causal therapy for HAE in the future. First positive results of CRISPR-based editing of the kallikrein B1 (KLKB1) gene in patients with HAE types I and II have been published. A single dose of NTLA-2002 led to a dose-dependent and sustained reduction in total plasma kallikrein levels, with no severe adverse events observed. Exploratory analyses showed a reduction in the number of HAE attacks per month at all dose levels [24]. 

## Authors’ contributions 

MF writing original draft, review and editing. BW review and editing. 

## Funding 

None. 

## Conflict of interest 

MF: personal: no conflicts of interest; institutional: IDCL participates in NIS PIQHAR (Prophylaxis Impact on Quality of life Impairment of HAE Patients with Lower Annual Base Attack Rates) 

BW: Lecture fees (mostly paid) from Ärztekammer Niedersachsen, ALK-Abelló, Bencard, Cogitando, BVDD, CSL Behring, DDG, DGA, DGAKI, DGP, fg-hno-aerzte, FOMF, NDG, NEDH e.V., Novartis, Roche Posay, Streamed Up, Takeda, ThermofisherScientific sowie Advisory Board (honoriert) von Biocryst, Biomarin, CSL Behring, Kalvista, Novartis, Sanofi-Aventis, Takeda. 

**Table 1.** Diagnosing hereditary angioedema.

Clinical symptoms/medical history	Recurrent peripheral swelling of the skin without urticaria/itching and no response to corticosteroids, epinephrine, or antihistamines			
	Abdominal pain attacks			
	Possible laryngeal swelling			
Family history	Possibly positive family history for HAE			
Laboratory findings	Reduced C4 and reduced C1-INH activity, with or without reduced C1-INH concentration in plasma			
		**C1-INH activity**	**C1-INH concentration**	**C4 concentration**
	HAE type I	↓	↓	↓
	HAE type II	↓	N/↑	↓

HAE = hereditary angioedema; C1-INH = C1 inhibitor; N = normal.

**Table 2.** On-demand treatment of acute hereditary angioedema attacks.

**Active ingredient/mode of action ** **(approved in Germany)**	**Way of administration**	**Dosage for children ** **by age or BW**	**Dosage for adolescents/adults**	**Special features**
**pdC1-INH (500/1,500) ** Berinert Plasma-derived C1-INH	IV	≥ 0 years: 20 IU/kg BW	20 IU/kg BW	Plasma product Reconstitution Home therapy/self-application Batch documentation
**pdC1-INH (500) ** Cinryze Plasma-derived C1-INH	IV	2 – 11 years (10 – 25 kg): 500 IU 2 – 11 years (> 25 kg): 1,000 IU	≥ 12 years (> 25 kg): 1,000 IU	Plasma product Reconstitution Home therapy/self-application Batch documentation
**rhC1-INH (2100) Conestat α** Recombinant analogue of the human C1-INH	IV	≥ 2 years (< 84 kg): 50 IU/kg BW	< 84kg: 50 IU/kg ≥ 84kg: 4,200 IU	Contains traces of rabbit protein Reconstitution Home therapy/self-application
**Icatibant ** Selective, competitive bradykinin type 2 receptor antagonist	SC	2 – 17 years • 12 – 25 kg: 10 mg (1 mL) • 26 – 40 kg: 15 mg (1.5 mL) • 41 – 50 kg: 20 mg (2 mL) • 51 – 65 kg: 25 mg (2.5 mL) • > 65 kg: 30 mg (3 mL)	≥ 18 years (> 65 kg): 30 mg (3 mL)	Prefilled syringe Home therapy/self-application

C1-INH = C1 inhibitor; IV = intravenously; SC = subcutaneously; BW = body weight. According to German product information. Of note (according to [3]): – Every attack in the head and neck area should be treated at any age! – Initiate therapy as early as possible! On-demand medication should always be readily available! – Treatment of children younger than 6 years of age should also include abdominal and peripheral attacks. – Consider treatment of attacks (outside the head and neck area, depending on severity) in children older than 6 years of age.

**Table 3.** Short-term prophylaxis prior to invasive medical procedures.

**Active ingredient/mode of action (approved in Germany)**	**Way of administration**	**Dosage for children by age or BW**	**Dosage for adolescents/adults**	**Special features**
**pdC1-INH (500/1,500) ** Berinert Plasma-derived C1-INH	IV	0 – < 18 years: 15 – 30 IU/kg BW	< 18 years: 15 – 30 IU/kg bw ≥ 18 years: 1,000 IU	Plasma product Reconstitution Home therapy/self-application Within 6 hours before the procedure
**pdC1-INH (500) ** Cinryze Plasma-derived C1-INH	IV	2 – 11 years (10 – 25 kg): 500 IU 2 – 11 years (> 25 kg): 1,000 IU	≥ 12 years (> 25 kg): 1,000 IU	Plasma product Reconstitution Home therapy/self-application Within 24 hours prior to the procedure

C1-INH = C1 inhibitor; IV = intravenously; BW = body weight. According to German product information. Of note (according to [3]): – Administration prior to all invasive medical procedures in the head/neck area, and as close as possible to the procedure! – If short-term prophylaxis is not used, on-demand therapy with C1-INH concentrate should be readily available.

**Table 4.** Long-term prophylaxis for the prevention of hereditary angioedema attacks.

**Active ingredient/mode of action (Approved in Germany)**	**Way of administration**	**Dosage for children by age or BW**	**Dosage for adolescents/adults**	**Special features**
**pdC1-INH (2000/3000) ** Berinert Plasma-derived C1-INH	SC	/	≥ 12 years: 60 IU/kg BW 2×/week	Plasma product Reconstitution Home therapy/self-application Batch documentation
**pdC1-INH (500**) Cinryze Plasma-derived C1-INH	IV	6 – 11 years: 500 IU every 3 – 4 days	≥ 12 years: 1,000 IU every 3 – 4 days	Plasma product Reconstitution Home therapy/self-application Batch documentation
**Lanadelumab ** (Recombinant human monoclonal antibody) Plasma kallikrein inhibitor	SC	2 – 11 years • 10 – ≤ 20 kg: 150 mg every 4 weeks • 20 – ≤ 40 kg: 150 mg every 2 weeks • ≥ 40 kg: 300 mg every 2 weeks	≥ 12 years: 300 mg every 2 weeks	Prefilled syringe Home therapy/self-application Dose adjustment possible Batch documentation
**Berotralstat ** (Small molecule) Synthetic oral plasma kallikrein inhibitor	PO	/	≥ 12 years (≥ 40 kg): 150 mg 1 × daily	Hard capsule Home therapy/self-application Consumption with a meal

C1-INH = C1 inhibitor; IV = intravenously; SC = subcutaneously; PO = orally; BW = body weight. According to German product information. Of note (according to [1, 3]): Consideration of the attack frequency (generally > 2 attacks per month), individual disease burden, as well as limitations in health-related quality of life and participation due to HAE, when deciding on long-term prophylaxis.
